# GTasm: a genome assembly method using graph transformers and HiFi reads

**DOI:** 10.3389/fgene.2024.1495657

**Published:** 2024-10-25

**Authors:** Junwei Luo, Ziheng Zhang, Xinliang Ma, Chaokun Yan, Huimin Luo

**Affiliations:** ^1^ School of Software, Henan Polytechnic University, Jiaozuo, China; ^2^ School of Computer and Information Engineering, Henan University, Kaifeng, China

**Keywords:** genome assembly, graph transformer, HiFi read, deep learning, sequencing technique

## Abstract

**Motivation:**

Genome assembly aims to reconstruct the whole chromosome-scale genome sequence. Obtaining accurate and complete chromosome-scale genome sequence serve as an indispensable foundation for downstream genomics analyses. Due to the complex repeat regions contained in genome sequence, the assembly results commonly are fragmented. Long reads with high accuracy rate can greatly enhance the integrity of genome assembly results.

**Results:**

Here we introduce GTasm, an assembly method that uses graph transformer network to find optimal assembly results based on assembly graphs. Based on assembly graph, GTasm first extracts features about vertices and edges. Then, GTasm scores the edges by graph transformer model, and adopt a heuristic algorithm to find optimal paths in the assembly graph, each path corresponding to a contig. The graph transformer model is trained using simulated HiFi reads from CHM13, and GTasm is compared with other assembly methods using real HIFI read set. Through experimental result, GTasm can produce well assembly results, and achieve good performance on NA50 and NGA50 evaluation indicators. Applying deep learning models to genome assembly can improve the continuity and accuracy of assembly results. The code is available from https://github.com/chu-xuezhe/GTasm.

## 1 Introduction

The genome sequence forms the foundation for research on species growth, development, morphology, disease, and lifespan. The completion of the Human Genome Project has provided us with a deeper understanding of genetic variations, disease treatment, and various aspects of human growth and development (Li et al., 2020; Wang T. et al., 2022). Genome assembly has become a significant issue in the field of genomic research. Genome assembly is the process of reconstructing a genome sequence from read set coming from different sequencing technologies (Sohn and Nam, 2018; Shyamli et al., 2021; Mwamburi et al., 2024). High-quality chromosome-scale genome sequences are an important basis for downstream studies in genomics.

The problem of complex and repetitive regions of genome sequence hinder genome assembly method from producing accurate results (Li and Durbin, 2024). Second-generation sequencing technology is characterized by high accuracy and high throughput, making it widely applicable in various fields of bioinformatics (Margulies et al., 2005; Satam et al., 2023). However, the main drawback of second-generation sequencing technology is that, the length of read it sequenced ranges approximately from 150 to 400 base pairs (bp) (Goodwin et al., 2016). The ability to assemble high-quality genome sequence has been dramatically improved with the advent and development of third-generation sequencing technologies. In contrast to short-read sequencing, long-read sequencing technologies can generate reads with thousands of base pairs in length, significantly simplifying the assembly process (Wang et al., 2023). Although long reads can span most repeat regions in genome sequence, its sequencing error rate usually is high (Wenger et al., 2019). In past few years, HiFi reads sequencing by the third generation sequencing technology are characterized by being both long and accurate. According to current standards for long-read sequencing, HiFi reads have a moderate length—often exceeding 50 kb. In contrast, ultra-long ONT data can exceed 100 kb, but ONT data generally has a higher error rate, typically over 5%. HiFi reads, on the other hand, are highly accurate, with a median accuracy exceeding 99.9% (>Q30) (Marks et al., 2019; Wenger et al., 2019; Zhang et al., 2022). Therefore, this study chooses to use HiFi reads for genome assembly.

Currently, *de novo* genome assembly mainly involves two types of method: De Bruijn graph-based methods (Idury and Waterman, 1995) and Overlap-Layout-Consensus (OLC) methods (Myers et al., 2000).

The De Bruijn graph-based assembly method is a widely utilized strategy in genome assembly, particularly effective for handling high-throughput short reads. This method involves fragmenting the DNA sequences from sequencing reads into fixed-length k-mers and constructing a De Bruijn graph using these k-mers to capture the overlapping relationships among reads. In the graph, each k-mer serves as a node, and the overlap between adjacent k-mers is represented as a directed edge. By traversing this graph, the original genome sequence can be efficiently reconstructed. This method demonstrates superior performance in handling repetitive regions within the genome and is capable of efficiently processing large-scale genomic data. However, selecting an appropriate k-mer length is crucial; a k value that is too small may result in an overly complex graph structure, while a k value that is too large may lead to graph discontinuities. Additionally, constructing and operating a De Bruijn graph typically requires substantial memory due to the need to store a large number of k-mers and edges. The De Bruijn graph-based method has become a cornerstone technique in modern genome assembly, finding broad application across data generated by various sequencing platforms (Huang and Liao, 2016). Flye (Lin et al., 2016; Kolmogorov et al., 2019; Kolmogorov et al., 2020) Canu (Koren et al., 2017) Verkko (Rautiainen et al., 2023) are good assembly methods based on De Bruijn graph method.

Flye is an efficient long-read genome assembly method specifically designed to handle PacBio and Oxford Nanopore reads. It employs a unique iterative overlap graph algorithm that enables the rapid generation of high-quality genome assembly results. Flye features robust error correction capabilities, significantly reducing the error rate in long-read data through a multi-step correction process, thereby improving assembly accuracy.

Canu is a high-accuracy genome assembly method specifically designed for handling high-noise long-read data from PacBio and Oxford Nanopore. HiCanu (Nurk et al., 2020) is an improved version of Canu, specifically optimized for the assembly of high-accuracy long-read data. Through multi-step error correction, overlap computation, assembly, and fine-tuning correction processes, it effectively reduces the error rate and enhances both the accuracy and continuity of the assembly.

Verkko is a new method designed for telomere-to-telomere (T2T) genome assembly. By integrating components such as Canu, MBG (Rautiainen and Marschall, 2021), GraphAligner (Rautiainen and Marschall, 2020), and Rukki, Verkko enables automated processing of input third-generation sequencing reads to achieve high-contiguity and high-accuracy haplotype-resolved genomes. With high-quality third-generation sequencing reads, Verkko can achieve T2T assembly level genomes.

The Overlap-Layout-Consensus (OLC) paradigm for sequence assembly is an overlap-based sequence assembly method. In this method, the input sequences are first aligned pairwise to find overlapping regions between them. Then, using these overlapping regions, the sequences are stitched together into a longer sequence, known as the layout. Then, the layout undergoes consensus analysis and correction to generate the final sequence (Pevzner et al., 2001). The OLC paradigm typically consists of three main steps: overlap detection, layout construction, and consensus analysis. This method works well for handling long sequences because it can fully leverage the overlap information between sequences, enhancing the accuracy and reliability of sequence assembly. The advantage of the OLC paradigm is its ability to handle long sequences and make full use of overlap information between sequences. Therefore, when dealing with sequence data that contains overlap information, it can typically produce accurate assembly results. However, the OLC method also faces some challenges, such as difficulties in handling highly repetitive sequences or large-scale datasets, and it has relatively high computational complexity. Hifiasm (Cheng et al., 2021; Cheng et al., 2022; Cheng et al., 2024) Raven (Vaser and Sikic, 2021) are good assembly methods based on OLC method.

Hifiasm uses high-precision long read for genome assembly, especially for PacBio HiFi data. Hifiasm takes advantage of the low error rate and high accuracy of HiFi reads, and employs highly efficient graph-structure construction and optimization algorithms to achieve fast and precise genome assembly. It is designed with the goal of providing high-quality genome assembly results, and excels especially when working with complex genomes and highly repetitive sequences.

Raven is an assembly method for *de novo* assembly using long reads, based on the overlap-layout-consensus (OLC) paradigm. It is specifically designed to handle high-noise long-read data from PacBio and Oxford Nanopore. It employs advanced assembly algorithms that can quickly and efficiently produce high-quality genome assembly results.

GNNome (Vrček et al., 2022; Vrček et al., 2024) is the first method to use deep learning methods to address the layout stage in sequence assembly. GNNome produces assembly results that surpass those of traditional assembly methods. GNNome uses Graph Convolutional Networks (GatedGCN) to score the edges in the assembly graph, and extract path to form contigs.

Although existing genome assembly methods have greatly improved the development of genomics research, we still need to further analyze the characteristics of long reads and develop new algorithms to resolve the problems caused by complex and repetitive regions. In De-Bruijn graph or assembly graph, complex and repetitive regions usually lead to more complex links, which is difficult to find accurate paths. Deep learning methods have demonstrated strong performance in solving problems that involve finding the correct path in complex graph structures. Although GNNome has adopted deep learning method for genome assembly, the Graph Convolutional Networks (GCNs) are effective for processing information from short-range neighboring vertices but are less effective at handling information from distant vertices. In this work, we use Graph Transformer network to further simultaneously process short-range and long-range relationship among vertices in assembly graph. And Graph Transformer network has achieved success in addressing the Traveling Salesman Problem (TSP) (Yang et al., 2023).

Here, we introduce a genome assembly method, GTasm, based on graph transformer model and assembly graph. GTasm can score edges in an assembly graph to identify the optimal path for reconstructing the genome sequence. The reads along the obtained path are assembled to produce the final assembly results. We construct a dataset using simulated data to train Graph Transformer (Vaswani et al., 2017; Dwivedi and Bresson, 2020; Yang et al., 2023). Compared to other assembly methods, GTasm can achieve well assembly results. Using the QUAST (Mikheenko et al., 2018) evaluation tool to assess the assembly results, GTasm has shown good performance in evaluation metrics such as misassemblies, NA50, and NGA50.

## 2 Methods

**FIGURE 1:**
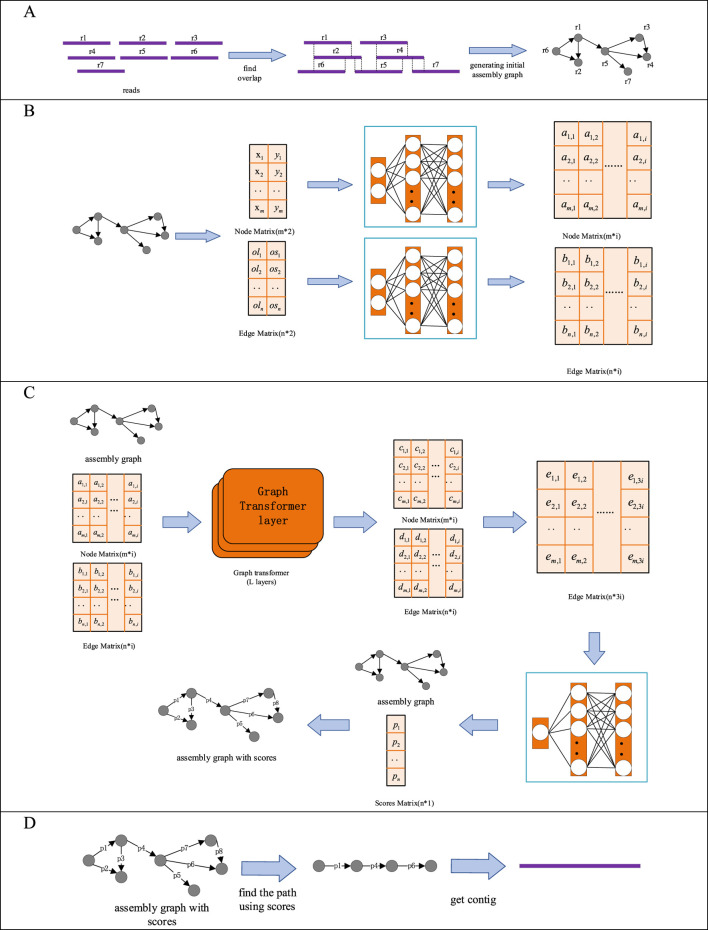
Detailed Processing Steps of GTasm. **(A)** Generating initial assembly graph. **(B)** Feature Extraction. **(C)** Scoring the edges in the assembly graph. **(D)** Obtaining assembly paths and contigs.

GTasm is a method for *de novo* genome assembly based on graph transformer model and HiFi reads. GTasm uses HiFi read set (FASTA OR FASTQ format) as input, and contig set (FASTA format) as output. GTasm primarily includes the following steps: (i) Generating initial assembly graph. The input HiFi read set is used to generate an assembly graph by Hifiasm, and the graph is stored in a GFA file. (ii) Feature Extraction. Node features and edge features are extracted from the initial assembly graph, and a fully connected layer is utilized to elevate the dimensionality of these features. (iii) Scoring the edges in the assembly graph. The generated initial assembly graph is converted into a DGL graph, which facilitates the processing of the graph using deep learning models. The converted DGL graph is Then input into the graph transformer model for scoring the edges in the initial assembly graph. (iv) Obtaining assembly paths and contigs. A greedy search algorithm is employed to search the assembly graph with edge scores, identifying assembly paths and assembling the reads along these paths to obtain the final contigs. The specific processing steps are illustrated in Figure 1.

### 2.1 Generating initial assembly graph

The first step of GTasm is to generate an initial assembly graph from the input set of reads. In the assembly graph, each vertex represents a long read, and the edges indicate the overlap relationships between corresponding long reads. The assembly graph is used to represent and analyze the overlaps among long reads, facilitating the correct connection and alignment of these long reads during genome assembly, ultimately constructing longer contiguous sequences (contigs) (Luo et al., 2021; Ding and Luo, 2022).

To construct the assembly graph, each pair of long reads is aligned to identify overlapping regions. The overlap length, starting position, and orientation are determined, and this information is used to create the edges in the assembly graph. The edge direction typically extends from the left end of the overlap to the right end, indicating that one long read overlaps with another in a specific direction. The assembly graph provides the foundation for subsequent assembly algorithms, and the quality of the initial assembly graph has a significant impact on the final assembly results. GTasm utilizes Hifiasm to generate the initial assembly graph (Gao et al., 2023).

### 2.2 Feature Extraction

After constructing the assembly graph *G*, GTasm extract the node features and edge features. For *i*th node $v_{i}$ , GTasm constructs its node feature vector [ $x_{i}$ , $y_{i}$ ], $x_{i}$ is the out-degree of $v_{i}$ , $y_{i}$ is the in-degree of $v_{i}$ . After getting features for all vertices, these feature vectors can form a matrix ${VM}_{m*2}$ , m is the number of the vertex in *G*, the *i*th row in ${VM}_{m*2}$ refers to the feature of $v_{i}$ . Similarly, we obtain the edge feature matrix ${EM}_{n*2}$ , where ${ol}_{i}$ is the overlap length of the *i*th edge, ${os}_{i}$ is the overlap similarity of the *i*th edge, and n denotes the number of edges in the assembly graph *G*. Edge features consist of the overlap length and overlap similarity between the nodes connected by the edge. The overlap length can be obtained from the CIGAR string in the initial GFA file. The GFA file stores the ID information of each read, chain information, base information of the reads, read lengths, information on which reads have overlaps, and the lengths of those overlaps. The method for calculating overlap similarity is as follows:

$$overlap\;similarity=\frac{overlap\;length-edit\;distance}{overlap\;length}$$

Edit Distance, also known as Levenshtein Distance, is a measure of the similarity between two strings. It represents the minimum number of edit operations required to transform one string into another. Common edit operations include inserting a character, deleting a character, and substituting one character for another. Edit Distance is calculated using the Edlib library (Šošić and Šikić, 2017). Then, GTasm uses three fully connected layers to transform the obtained node features and edge features into higher-dimensional representations, resulting in the node feature matrix ${VM}_{m*i}$ and the edge feature matrix ${\;EM}_{m*i}$ ,where *i* = 64, The activation function used is ReLU.

### 2.3 Scoring the edges in the assembly graph

**FIGURE 2:**
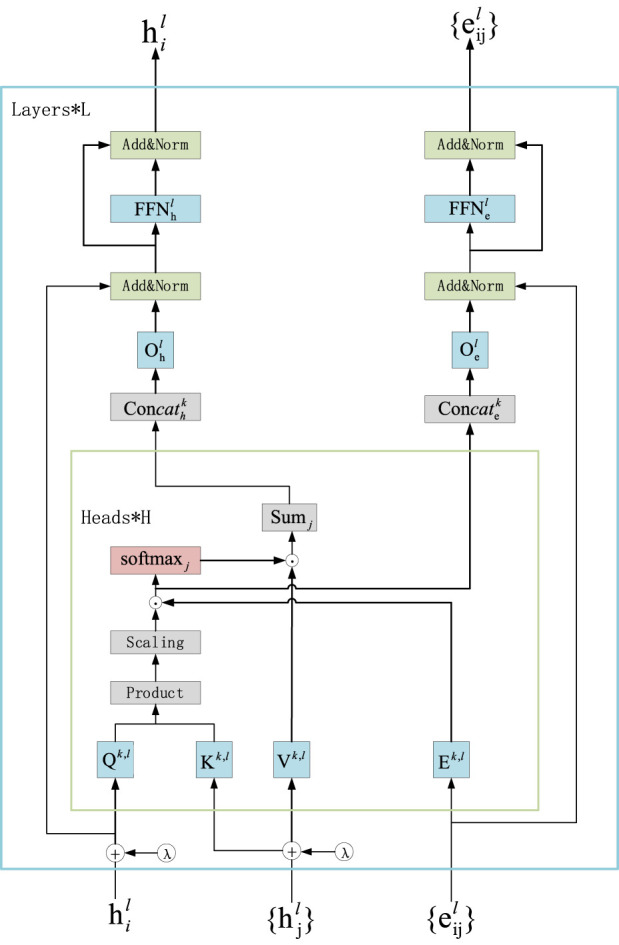
Network structure diagram of the transformer. The Graph Transformer framework uses Laplacian eigenvectors (λ) as positional encodings (LapPE). LapPE is added to the input node embeddings before passing the features into the first layer. The Graph Transformer operates on node features and edge features to compute attention scores.

Scoring the edges in the assembly graph is a core step in the entire processing flow. We conduct subsequent searches for assembly paths based on the probability scores of the edges in the assembly graph. The core problem we aim to address is finding an optimal assembly path in the assembly graph to achieve the best assembly result. Since this is a graph-related problem, it naturally leads us to consider using graph neural networks (GNNs) to tackle the issue, as GNNs have proven to be an effective neural network architecture for graph datasets. Transformers have achieved great success in the field of natural language processing (NLP). They excel in handling long-range sequence issues, which is achieved through the use of attention mechanisms. Graph transformers are the application of transformers in graph neural networks, allowing them to focus on the relationships between distant nodes in the graph and demonstrating strong performance in solving graph-related problems (Yun et al., 2022; Boadu et al., 2023; Cai et al., 2023; Corrias et al., 2023; Zhao et al., 2023). Using the graph transformer model to score the edges in the initial assembly graph helps address the issue of repetitive regions in genome assembly. The graph transformer model can utilize multi-head attention mechanisms to compute the importance weights of the edges in the graph, effectively processing information from distant nodes to uncover relationships between nodes that are not directly connected. The main structure of the Graph Transformer layer is shown in Figure 2.

The graph information and extracted node feature matrix ${VM}_{m*i}$ and edge feature matrix ${EM}_{n*i}$ are fed into a Graph Transformer layer for processing, with the Graph Transformer layer preserving the dimensionality of the feature matrices. The processed node and edge feature matrices are then used to update the edge feature matrix. Specifically, the features of the source and target nodes connected by each edge are combined with the edge’s own features to generate a new edge feature matrix ${EM}_{m*3i}$ .This updated edge feature matrix ${EM}_{m*3i}$ is then passed through a three-layer Multi-Layer Perceptron (MLP) to produce a score matrix ${SM}_{n*1}$ (Savalia and Emamian, 2018).The values in the score matrix ${SM}_{n*1}$ correspond to the probability scores of each edge in the assembly graph *G*. These scores are then written back into the assembly graph, resulting in an assembly graph annotated with edge probability scores. Next, we introduce the process of Graph Transformer model to obtain the probability scores of edges in the assembly graph. First, we input the processed node features and edge features into the Graph Transformer. The update formula for the l-th layer is as follows:

$${\hat{h}}_{i}^{l+1}=O_{h}^{l}\overset{H}{\underset{k=1}{\|}}\left(\sum_{j\in N_{i}}w_{ij}^{k,l}V^{k,l}h_{j}^{l}\right)$$

$${\hat{e}}_{ij}^{l+1}=O_{e}^{l}\overset{H}{\underset{k=1}{\|}}\left({\hat{w}}_{ij}^{k,l}\right)$$

$$w_{ij}^{k,l}=soft\max_{j}\left({\hat{w}}_{ij}^{k,l}\right)$$

$${\hat{w}}_{ij}^{k,l}=\left(\frac{Q^{k,l}h_{i}^{l}\cdot K^{k,l}h_{j}^{l}}{\sqrt{d_{k}}}\right)\cdot E^{k,l}e_{ij}^{l}$$

Where, $Q^{k,l},K^{k,l},V^{k,l},E^{k,l}\in R^{d_{k}\times d},O_{h}^{l},O_{e}^{l}\in R^{d\times d}$ , *k* = 1 to *H* represents the number of attention heads, and || denotes concatenation, h and e are the node and edge features input to the graph Transformer, while *Q*, *K*, and *V* are the parameters used to compute the attention scores. For numerical stability, the outputs of the SoftMax function are clamped to the range of −5 to +5 after exponentiation. The attention output is then passed to the feed-forward network (FFN), with residual connections and normalization layers applied before and after the FFN.

h^^l+1=Normhil+h^il+1

h^^^il+1=W2lReLUW1lh^^il+1

hil+1=Normh^^il+1+h^^^il+1

where $W_{e,1}^{l}\in R^{2d\times d},W_{e,2}^{l}\in R^{d\times 2d},{\hat{\hat{e}}}_{ij}^{l+1},{\hat{\hat{\hat{e}}}}_{ij}^{l+1}$ represents intermediate representations, and Norm refers to Batch Normalization.

e^^ijl+1=Normeijl+e^ijl+1

e^^^ijl+1=We,2lReLUWe,1le^^ijl+1

eijl+1=Norme^^ijl+1+e^^^ijl+1

where We,1l∈R2d×d,We,2l∈Rd×2d,e^^ijl+1,e^^^ijl+1 represents intermediate representations, and Norm refers to Batch Normalization.

After getting edge matrix, for each directed edge *s→t*, its feature vector refers to one row in the edge matrix, the formula for calculating its probability score $p_{st}$ is:

$$p_{st}=\sigma \left(MLP\left(EM_{n*3i}\right)\right)$$

where σ is the sigmoid function.

### 2.4 Obtaining assembly paths and contigs

GTasm runs an iterative greedy search algorithm on the assembly graph with edge probability scores. Our greedy search algorithm has lower space complexity, making it more suitable for resource-constrained environments. Specifically, we first randomly select the *N* edges with the highest probability scores from the assembly graph. Then, starting from these *N* edges, we perform forward and backward greedy searches sequentially, connecting the paths obtained from forward and backward traversals to form paths starting from these edges. We then mark the nodes that have already been traversed to prevent them from being revisited. After *N* rounds of traversal, we obtain *N* paths. The reads from the nodes in each path are then concatenated to form a contig for each path. We then select the longest contig among these *N* contigs as our contig and mark the nodes in this contig’s path to prevent them from being revisited. This process is repeated by selecting new *N* starting edges to obtain new contigs, and the iteration continues until the length of the obtained paths falls below a certain threshold. After obtaining all the assembly paths, GTasm extracts the nodes along the paths and records each node’s read length and overlap length in the GFA file. Using the read lengths and overlap lengths with other reads, the reads along the assembly paths can be concatenated to form the contig for that specific assembly path.

### 2.5 Model training

#### 2.5.1 Dataset

We train Graph Transformer using HiFi reads. HiFi reads are characterized by being both long and highly accurate, with median read accuracy exceeding 99.9% (>Q30). For training and validation, we use a simulated dataset generated from the CHM13 (Nurk et al., 2022) human genome sequence. Real HG002 HiFi read datasets are used for testing.

The generation of the simulated reads is inspired by the approach used in GNNome. First, the CHM13 reference sequence is obtained and then divided into 23 chromosomes: chromosomes 1 to 22 and the X chromosome. Next, simulated reads are generated from the chromosomes using a read simulator pbsim3 (Ono et al., 2022). We adopt pbsim3 to generate simulated reads using CHM13 reference sequence and CHM13 real read dataset. The simulated reads are annotated with their positions relative to the reference sequence, which helps us label the assembly graph based on the relative positions of the reads. By using the relative positions of the reads, we can easily determine if there are overlaps between reads, allowing us to label the edges in the assembly graph.

We generate simulated reads in the form of single chromosomes, allowing us to easily divide the data into training and validation sets using individual chromosomes. Each simulation generates different read sets, resulting in different assembly graphs. This ensures a sufficient amount of data for training the Graph Transformer, which is why we use simulated reads for training rather than real reads. We simulated reads for chromosomes 1 to 22 of CHM13. Chromosomes 1, 3, 5, 7, 9, 11, 13, 15, 17, 19, and 21 were used as the training set, while chromosomes 2, 4, 6, 8, 10, 12, 14, 16, 18, 20, and 22 were used as the validation set. For the training set, seven simulated read sets were generated for each chromosome. For the validation set, three simulated read sets were generated for each chromosome. The test set uses real datasets. The selection of chromosomes for the training and validation sets, as well as the number of datasets generated for each chromosome, was done entirely at random. The details about all datasets are shown in Supplementary Table S1 of the Supplementary Material.

After generating simulated reads for the chromosomes, we use Hifiasm to create assembly graphs from these simulated reads. Then, we label the generated assembly graphs. Each read in the simulated datasets is annotated with information about its start position, end position, and strand orientation relative to the reference sequence. We perform an initial simplification of the assembly graph. If two reads have the same strand orientation and overlap, we retain the corresponding edge. Edges that do not meet this criterion are removed from the assembly graph. Specifically, if there is a directed edge $e_{ab}$ from node A to node B, and the following conditions are met: The start position of node A is less than the start position of node B. The end position of node A is greater than the start position of node B. The end position of node B is greater than the end position of node A. Then, the directed edge $e_{ab}$ is retained.

In the retained edges, there may be some tips. To remove these tips, first identify the node with the lowest start position relative to the reference sequence. Perform a breadth-first search (BFS) starting from this node (Li, 2016). Then, select the node with the highest position among all visited nodes and perform a backward breadth-first search starting from this node. If an edge is traversed both forward and backward, it is marked as positive; otherwise, it is marked as negative. Repeat this process until all edges are labeled.

#### 2.5.2 Training

Set a random seed during training to ensure the reproducibility of experiments. Graph Transformer is trained using the Adam optimizer (Tang et al., 2021). Due to the extreme imbalance between positive and negative samples in the dataset, we calculate the ratio of positive to negative samples to balance the weights during scoring. We use the simulated CHM13 dataset, which consists of HiFi reads with 32× coverage depth, as the training and validation sets. Even for a single chromosome, the generated assembly graph has tens of thousands of nodes and hundreds of thousands of edges. This large graph imposes high computational resource requirements for direct training. Therefore, we use the METIS (Mitchell et al., 2005) graph partitioning algorithm to divide the assembly graph for each chromosome into several subgraphs, and perform training on these subgraphs. The learning objective of the model is a binary edge classification task. During training, we compute the binary cross-entropy loss between the model’s predictions and the labels. The formulation is expressed as follows:

$$Loss=BCE\left(p,edge\_label\right)$$

where BCE represents binary cross-entropy, and p denotes the probability score computed after processing through the Graph Transformer. The initial learning rate is set to 1e-4. We use pytorch 1.7 and DGL 2.1 (Wang, 2019) to implement the network. The server is equipped with an NVIDIA RTX 4090 GPU, an Intel(R) Xeon(R) Gold 6330 CPU @ 2.00GHz, and 512 GB of RAM.

## 3 Results

We evaluate GTasm, GNNome, Hifiasm, Flye, Raven, Verkko, and HiCanu on HiFi datasets from the human genome CHM13, the human genome HG002 (Wang T. et al., 2022), the inbred genome of A. thaliana, and the maternal genome of G. gallus (Wang B. et al., 2022). The command lines of all methods are supplied in the Supplementary Material.

We used the QUAST evaluation tool to assess the assembly results, comparing and evaluating them using the following metrics: Contigs, Largest contig, Misassemblies, Genome fraction, NA50, and NGA50. Contigs indicate the number of contigs in the assembly results, while Largest contig refers to the length of the longest contig. Misassemblies refer to errors in the genome assembly process that leads to discrepancies between the assembled genome sequence and the actual genome sequence. These errors can arise from various causes, including sequencing errors, repetitive sequences, and insufficient coverage. Genome fraction represents the proportion of the reference genome covered by the assembled sequences. NA50 and NGA50 are important metrics for assessing genome assembly quality. NA50 is based on the alignment of the assembly sequences to the reference genome. NA50 is calculated based on the alignment results of the genome assembly. The calculation method involves aligning the assembled contigs to the reference genome and sorting the aligned blocks by length in descending order. The NA50 value is the length of the shortest block such that the cumulative length of these blocks is at least 50% of the total length of the assembly result. It reflects the continuity of aligned blocks within the assembly. NGA50 is calculated based on the length of the reference genome. The calculation method involves aligning the assembled contigs to the reference genome and sorting the aligned blocks by length in descending order. The NGA50 value is the length of the shortest block such that the cumulative length of these blocks is at least 50% of the length of the reference genome. It reflects the coverage of aligned blocks within the reference genome, taking into account gaps in the alignments.

As shown in Table 1, we can see that GTasm demonstrates well continuity, as evidenced by the NG50 and NGA50 metrics. On the human CHM13 HiFi dataset, GTasm’s NG50 and NGA50 metrics are better than those of HiCanu, nearly twice those of Flye, and almost three times those of Raven and Verkko. Compared to Hifiasm and GNNome, GTasm also shows superior performance in terms of continuity. In terms of misassemblies, Verkko performs the best, which can be attributed to its built-in polishing process. GTasm shows similar performance to GNNome but is slightly inferior to Hifiasm. Verkko, Flye, and HiCanu all have built-in polishing processes, which contribute to their strong performance on the misassemblies metric, although their runtime is correspondingly longer. The runtime and memory consumption of each method are shown in Table 5. For genome fraction, all seven methods perform well, with Hifiasm, GNNome, Verkko, HiCanu, and GTasm all achieving over 99.5%.

**TABLE 1:** Results on real CHM13 HiFi data.

Dataset	SW	Contigs	LC (Mbp)	Mis	GF (%)	NA50 (Mbp)	NGA50 (Mbp)
CHM13	Hifiasm	385	**201.07**	132	99.646	67.92	67.92
	GNNome	**119**	160.74	174	99.592	63.72	63.72
	Flye	1,461	114.66	266	97.775	36.32	35.41
	Raven	1945	103.48	1,519	97.17	22.13	21.94
	Verkko	2,112	109.34	**93**	**99.8**	26.04	26.04
	HiCanu	7,976	142.32	185	99.683	41.48	47.40
	GTasm	195	191.34	174	99.681	**70.83**	**70.83**

Note: DS, dataset; SW, software; LC, Largest contig.; Mis, Misassemblies.

The bold items in the table indicate the optimal results for that metric.

Table 2 shows the test results on the HG002 dataset, GTasm also demonstrates strong performance. In terms of misassemblies, HiCanu shows the best results, while Flye also performs well. Raven, which lacks a polishing step, performs the worst, highlighting the importance of polishing for assembly accuracy. For assembly completeness, Verkko achieves the best performance, with a genome fraction of 92.808%. GTasm and GNNome perform similarly and both outperform Hifiasm, while significantly exceeding the results of other methods. Notably, Raven assembled only half the length of the reference genome. In terms of continuity, GTasm has a slightly lower NA50 compared to Hifiasm but a higher NGA50, showing similar results to GNNome. Compared to Flye, Verkko, and HiCanu, GTasm excels in continuity, with nearly six times higher NA50 and NGA50 than Verkko, despite similar misassemblies. Although Raven has the highest NA50, its poor assembly completeness led to QUAST not calculating an NGA50 value. Overall, GTasm significantly outperforms other traditional assembly methods in terms of continuity.

**TABLE 2:** Results on real HG002 HiFi data.

Dataset	SW	Contigs	LC (Mbp)	Mis	GF (%)	NA50 (Mbp)	NGA50 (Mbp)
HG002	Hifiasm	5,660	138.38	1,009	73.036	2.64	1.11
	GNNome	4,727	**138.99**	1,449	84.317	2.17	2.01
	Flye	29,666	40.40	880	64.013	0.38	0.11
	Raven	**3,923**	45.10	3,709	51.449	**3.69**	—
	Verkko	58,926	45.24	1,513	**92.808**	0.27	0.28
	HiCanu	30,081	121.36	**869**	81.873	0.89	0.93
	GTasm	4,676	104.70	1705	84.517	2.00	1.86

Note: The bold items in the table indicate the optimal results for that metric

Table 3 shows the test results on the non-human dataset A. thaliana, GTasm performs well. GTasm generates the fewest contigs and the longest contig length. For misassemblies, Flye shows the best performance, far surpassing other methods. GTasm’s performance is second only to Flye and Raven, with fewer errors compared to other methods. In terms of genome fraction, all seven methods perform well, with Flye at 98.8%, Raven at 99.5%, and the remaining methods achieving results above 99.9%. GTasm has the best performance in continuity. In NA50 values, GTasm is nearly 6 times better than Verkko and nearly twice as good as Hifiasm, Flye, Raven, and HiCanu, showing even better results compared to GNNome. In NGA50 values, GTasm achieves results similar to Hifiasm and GNNome, and significantly outperforms Flye, Raven, Verkko, and HiCanu. Overall, GTasm demonstrates the best performance on the A. thaliana dataset.

**TABLE 3:** Results on real A.thaliana HiFi data.

Dataset	SW	Contigs	LC (Mbp)	Mis	GF (%)	NA50 (Mbp)	NGA50 (Mbp)
A.thaliana	Hifiasm	1724	**22.93**	131	**99.988**	6.99	12.75
	GNNome	98	**22.93**	147	99.987	8.64	12.75
	Flye	99	15.94	**35**	98.868	6.39	6.39
	Raven	179	11.49	113	99.565	5.08	5.19
	Verkko	3,809	12.70	238	99.966	2.56	6.01
	HiCanu	1,587	**22.93**	154	99.943	6.39	8.57
	GTasm	**92**	**22.93**	124	99.957	**12.75**	**12.75**

Note: The bold items in the table indicate the optimal results for that metric

Table 4 shows the assembly results of different methods on G. gallus HiFi reads. For misassemblies, Flye performs the best, while GTasm performs poorly. In terms of genome fraction, the methods exhibit similar performance. In continuity, GTasm has the best results. GTasm shows better NA50 and NGA50 values than Hifiasm. For NA50 and NGA50 values, GTasm is nearly 5 times better than Verkko and Flye, and nearly twice as good as Raven. HiCanu crashed after running for 10 days, and no results were obtained. Overall, GTasm and GNNome show similar performance on the G. gallus data.

**TABLE 4:** Results on real G.gallus HiFi data.

Dataset	SW	Contigs	LC (Mbp)	Mis	GF (%)	NA50 (Mbp)	NGA50 (Mbp)
G.gallus	Hifiasm	1866	**47.52**	7,241	**98.516**	1.61	3.09
	GNNome	**1,516**	28.16	7,443	98.239	2.03	4.20
	Flye	7,463	10.54	**5,151**	98.056	0.47	0.91
	Raven	2,117	14.06	5,343	96.801	1.32	1.54
	Verkko	9,156	3.62	6,027	97.176	0.47	0.93
	HiCanu	—	—	—	—	—	—
	GTasm	1,534	31.46	7,644	98.229	**2.04**	**4.21**

Note: The bold items in the table indicate the optimal results for that metric

In summary, GTasm demonstrates well results across all four human and non-human datasets. The Graph Transformer can capture relationships between distant nodes, which is why we observe GTasm performing better in terms of continuity on most datasets.

Table 5 records the time and memory consumption of different methods across various datasets. For GNNome and GTasm, since they require the assembly graph generated by Hifiasm as a prerequisite, the recorded time includes the time spent running Hifiasm. If the memory required by subsequent steps of these methods is less than that consumed by Hifiasm, the memory usage of Hifiasm is recorded as their memory requirement. The server configuration for running the above methods is as follows: CPU: Intel(R) Xeon(R) Gold 6330 CPU @ 2.00GHz, with 512 GB of RAM.

**TABLE 5:** Time and memory for different datasets.

Dataset	Software	Time (H:M:S)	Memory (GB)
CHM13	Hifiasm	9:56:10	126.32
	GNNome	15:8:46	126.32
	Flye	20:56:59	151.62
	Raven	9:55:46	96.13
	Verkko	56:21:20	73.54
	HiCanu	53:54:11	96.43
	GTasm	16:07:32	126.32
HG002	Hifiasm	8:40:44	153.09
	GNNome	47:17:1	179.50
	Flye	22:43:41	203.88
	Raven	9:16:53	106.59
	Verkko	54:02:29	28.64
	HiCanu	60:29:45	73.34
	GTasm	48:13:27	179.50
A.thaliana	Hifiasm	6:21:09	52.30
	GNNome	6:37:23	52.30
	Flye	5:45:39	61.00
	Raven	3:09:24	49.55
	Verkko	16:06:41	21.33
	HiCanu	41:14:17	19.91
	GTasm	6:53:12	52.30
G.gallus	Hifiasm	2:42:07	50.29
	GNNome	5:03:37	50.29
	Flye	7:35:12	111.84
	Raven	2:40:15	53.08
	Verkko	8:41:20	19.05
	HiCanu	—	—
	GTasm	5:33:17	50.29

## 4 Conclusion and discussion

In this study, we developed a deep learning-based genome assembly method. GTasm extracts node and edge features from the assembly graph and uses Graph Transformer to analyze these features. The processed features are then scored using a three-layer MLP, which assigns scores to the edges in the assembly graph. These scores are used to traverse the graph and obtain assembly paths and contigs. We evaluated GTasm’s performance on multiple datasets and compared it with six state-of-the-art methods. GTasm demonstrated good performance across different HiFi read datasets.

In this paper, our work focuses on scoring the edges in the assembly graph and using a search algorithm to obtain assembly paths and contigs. The quality of the initial assembly graph has a significant impact on the final assembly results, and in this study, we have not yet completed the independent generation of the assembly graph. DNA sequences contain rich information for genome assembly, which we have not yet effectively utilized. We will address these issues in the future work.

## Data Availability

The CHM13 HiFi dataset used in this study can be accessed at https://github.com/marbl/CHM13. The HG002 HiFi dataset is available at https://github.com/human-pangenomics/HG002_Data_Freeze_v1.0. The A. thaliana HiFi dataset can be found at https://ngdc.cncb.ac.cn/gsa/browse/CRA004538/CRX257574. The G. gallus HiFi dataset is available at https://www.genomeark.org/genomeark-all/Gallus_gallus.html. Download link for the CHM13 reference sequence: https://s3-us-west-2.amazonaws.com/human-pangenomics/T2T/CHM13/assemblies/chm13.draft_v1.1.fasta.gz. Download link for the HG002 reference sequence: https://s3-us-west-2.amazonaws.com/human-pangenomics/T2T/HG002/assemblies/hg002v0.7.fasta. Download link for the A. thaliana reference sequence: https://download.cncb.ac.cn/gwh/Plants/Arabidopsis_thaliana_AT_GWHBDNP00000000.1/GWHBDNP00000000.1.genome.fasta.gz. Download link for the G. gallus reference sequence: https://www.ncbi.nlm.nih.gov/datasets/genome/GCF_016699485.2/.
