# Supplementary material for: GTasm: a genome assembly method using graph transformers and HiFi reads
Source: Front Genet. 2024 Oct 25;15:1495657. doi: 10.3389/fgene.2024.1495657 (PMC11543488; doi:10.3389/fgene.2024.1495657)
Supplement: Supplementary file 1 [file Table1.DOCX]

Supplementary Materials

## Dataset information

Table S1. Information about the datasets used in the paper

| Dataset | TRL(bp) | ARL(bp) | RSL(bp) | Coverage depth |
| --- | --- | --- | --- | --- |
| CHM13-sim-chr1 | 7,948,397,462 | 20,713.60 | 248387328 | 32× |
| CHM13-sim-chr2 | 7,766,316,813 | 20,714.60 | 242696752 | 32× |
| CHM13-sim-chr3 | 6,435,398,399 | 20,709.60 | 201105948 | 32× |
| CHM13-sim-chr4 | 6,194,403,514 | 20,709.10 | 193574945 | 32× |
| CHM13-sim-chr5 | 5,825,470,871 | 20,716.90 | 182045439 | 32× |
| CHM13-sim-chr6 | 5,508,057,660 | 20,714.80 | 172126628 | 32× |
| CHM13-sim-chr7 | 5,138,168,409 | 20,721.20 | 160567428 | 32× |
| CHM13-sim-chr8 | 4,680,306,920 | 20,717.60 | 146259331 | 32× |
| CHM13-sim-chr9 | 4,819,768,625 | 20,719.70 | 150617247 | 32× |
| CHM13-sim-chr10 | 4,312,261,842 | 20,705 | 134758134 | 32× |
| CHM13-sim-chr11 | 4,324,105,967 | 20,714.50 | 135127769 | 32× |
| CHM13-sim-chr12 | 4,266,393,604 | 20,722.90 | 133324548 | 32× |
| CHM13-sim-chr13 | 3,634,148,183 | 20,714.70 | 113566686 | 32× |
| CHM13-sim-chr14 | 3,237,179,999 | 20,719.10 | 101161492 | 32× |
| CHM13-sim-chr15 | 3,192,103,167 | 20,713.40 | 99753195 | 32× |
| CHM13-sim-chr16 | 3,082,573,235 | 20,720.80 | 96330374 | 32× |
| CHM13-sim-chr17 | 2,696,877,433 | 20,728.60 | 84276897 | 32× |
| CHM13-sim-chr18 | 2,577,366,903 | 20,717.60 | 80542538 | 32× |
| CHM13-sim-chr19 | 1,974,641,240 | 20,717.20 | 61707364 | 32× |
| CHM13-sim-chr20 | 2,118,747,454 | 20,715.40 | 66210255 | 32× |
| CHM13-sim-chr21 | 1,442,907,203 | 20,714.50 | 45090682 | 32× |
| CHM13-sim-chr22 | 1,642,412,309 | 20,712.90 | 51324926 | 32× |
| CHM13 | 100,369,168,661 | 18,028.80 | 3054832041 | 32.86× |
| HG002 | 110,549,151,396 | 14,971.10 | 5999588638 | 18.43× |
| A.thaliana | 22,904,700,074 | 15,094.40 | 133725193 | 171.28× |
| G.gallus | 33,945,723,745 | 22,754 | 1053332251 | 32.22× |

TRL: Total read length; ARL: Average read length; RSL: Reference sequence length;

## Table S2. Software information

| software | version | link |
| --- | --- | --- |
| Hifiasm | 0.18.8 | https://github.com/chhylp123/hifiasm |
| GNNome | - | https://github.com/lbcb-sci/GNNome |
| Flye | 2.9.4-b1799 | https://github.com/mikolmogorov/Flye |
| Raven | 1.83 | https://github.com/lbcb-sci/raven |
| Verkko | 2.0 | https://github.com/marbl/verkko |
| HiCanu | 2.2 | https://github.com/marbl/canu |

Note GNNome does not have version information labeled.

## Table S3 Detailed time and memory usage

| Dataset | Software | Step1 | | Step2 | | Step3 | |
| --- | --- | --- | --- | --- | --- | --- | --- |
|  |  | Time | Memory(GB) | Time | Memory(GB) | Time | MemoryGB) |
| CHM13 | GNNome | 9:56:10 | 126.32 | 3:49:56 | 123.26 | 1:22:40 | 83.0 |
|  | GTasm | 9:56:10 | 126.32 | 3:49:56 | 123.26 | 2:21:26 | 76.83 |
| HG002 | GNNome | 8:40:44 | 153.09 | 3:37:44 | 179.50 | 35:58:33 | 123.61 |
|  | GTasm | 8:40:44 | 153.09 | 3:37:44 | 179.50 | 36:54:59 | 112.77 |
| A.thaliana | GNNome | 6:21:09 | 52.30 | 0:13:18 | 13.49 | 0:2:56 | 10.79 |
|  | GTasm | 6:21:09 | 52.30 | 0:13:18 | 13.49 | 0:18:45 | 9.46 |
| G.gallus | GNNome | 2:42:7 | 50.29 | 0:32:37 | 43.07 | 1:48:53 | 3.81 |
|  | GTasm | 2:42:7 | 50.29 | 0:32:37 | 43.07 | 2:18:33 | 28.63 |

Step 1 indicates the generation of the initial assembly graph using Hifiasm. Step 2 represents the conversion of the assembly graph into a DGL graph. Step 3 involves solving the graph to obtain the assembly results.

## Commands Lines:

**Hifiasm:**

The command used to generate assembly graphs and results with Hifiasm is:

hifiasm --prt-raw -o <outputPrefix> -t <nThreads> -l0 <HiFi reads>

**GNNome:**

The commands used to generate assembly results with GNNome are:

1. Use Hifiasm command to generate assembly graphs.

2. Generate DGL graph:

python create_inference_graphs.py --reads <HiFi reads> --gfa <outputPrefix>.bp.p_ctg.gfa --asm hifiasm --out <out path>

3. Generate assembly results:

python inference.py --data <out path> --asm hifiasm --out <out path>

**Flye:**

The command used to generate assembly results with Flye is:

flye --pacbio-hifi <HiFi reads> --out-dir <out path> -t <nThreads>

**Raven:**

The command used to generate assembly results with Raven is:

raven --identity 0.99 -k29 -w9 -t64 -p0 <HiFi reads> > <outputPrefix>

**Verkko:**

The command used to generate assembly results with Verkko is:

verkko -d <out path> --hifi <HiFi reads> --local-cpus <nThreads>

**Hicanu:**

The command used to generate assembly results with HiCanu is:

canu -p asm -d <out path> genomeSize=<Gsize> maxThreads=<nThreads> -pacbio-hifi <HiFi reads>
